# Predicting risk of mortality in dialysis patients: a retrospective cohort study evaluating the prognostic value of a simple chest X-ray

**DOI:** 10.1186/1471-2369-14-263

**Published:** 2013-12-01

**Authors:** Ethan Bohn, Navdeep Tangri, Brent Gali, Blair Henderson, Manish M Sood, Paul Komenda, Claudio Rigatto

**Affiliations:** 1 University of Manitoba, Winnipeg, Canada; 2 Renal Health Program, Seven Oaks General Hospital, 2PD02 – 2300 McPhillips Street, Winnipeg, Manitoba R2V 3 M3, Canada; 3 Department of Radiology, Seven Oaks General Hospital, 2PD02 – 2300 McPhillips Street, Winnipeg, Manitoba R2V 3 M3, Canada

**Keywords:** Cardiovascular disease, Dialysis, Prognostication, Retrospective cohort, X-ray

## Abstract

**Background:**

Clinical outcomes of dialysis patients are variable, and improved knowledge of prognosis would inform decisions regarding patient management. We assessed the value of simple, chest X-ray derived measures of cardiac size (cardiothoracic ratio (CTR)) and vascular calcification (Aortic Arch Calcification (AAC)), in predicting death and improving multivariable prognostic models in a prevalent cohort of hemodialysis patients.

**Methods:**

Eight hundred and twenty-four dialysis patients with one or more postero-anterior (PA) chest X-ray were included in the study. Using a validated calcification score, the AAC was graded from 0 to 3. Cox proportional hazards models were used to assess the association between AAC score, CTR, and mortality. AAC was treated as a categorical variable with 4 levels (0,1,2, or 3). Age, race, diabetes, and heart failure were adjusted for in the multivariable analysis. The criterion for statistical significance was p<0.05.

**Results:**

The median CTR of the sample was 0.53 [IQR=0.48,0.58] with calcification scores as follows: 0 (54%), 1 (24%), 2 (17%), and 3 (5%). Of 824 patients, 152 (18%) died during follow-up. Age, sex, race, duration of dialysis, diabetes, heart failure, ischemic heart disease and baseline serum creatinine and phosphate were included in a base Cox model. Both CTR (HR 1.78[1.40,2.27] per 0.1 unit change), area under the curve (AUC)=0.60[0.55,0.65], and AAC (AAC 3 vs 0 HR 4.35[2.38,7.66], AAC 2 vs 0 HR 2.22[1.41,3.49], AAC 1 vs 0 HR 2.43[1.64,3.61]), AUC=0.63[0.58,0.68]) were associated with death in univariate Cox analysis. CTR remained significant after adjustment for base model variables (adjusted HR 1.46[1.11,1.92]), but did not increase the AUC of the base model (0.71[0.66,0.76] vs. 0.71[0.66,0.76]) and did not improve net reclassification performance (NRI=0). AAC also remained significant on multivariable analysis, but did not improve net reclassification (NRI=0). All ranges were based on 95% confidence intervals.

**Conclusions:**

Neither CTR nor AAC assessed on chest x-ray improved prediction of mortality in this prevalent cohort of dialysis patients. Our data do not support the clinical utility of X-ray measures of cardiac size and vascular calcification for the purpose of mortality prediction in prevalent hemodialysis patients. More advanced imaging techniques may be needed to improve prognostication in this population.

## Background

Kidney failure is a major public health problem with increasing incidence and prevalence worldwide [1]. Patients with kidney failure on dialysis experience poor overall survival, with an age and sex adjusted mortality several fold higher than patients not on dialysis [2]. Although aggregate survival on dialysis is poor, variability in individual patient prognosis is substantial [3]. This poses significant challenges for health care providers and patients alike. Survival estimates are a crucial part of informed discussions regarding starting or withdrawing from dialysis, and often inform decisions regarding the intensity of screening, monitoring and treatment of comorbid diseases and referral for kidney transplant [4-6]. Uncertainty about these outcomes can render such decisions more difficult for patients, families, and physicians.

In order to estimate survival, knowledge of risk factors is essential. Mortality in dialysis is driven primarily by cardiovascular (CV) disease [7,8]. Consequently, major factors associated with cardiovascular disease on dialysis, such as left ventricular hypertrophy (LVH) and coronary or aortic calcification, are independent predictors of mortality and cardiovascular events in dialysis patients [9,10]. However, the high cost and unknown benefit of risk stratification based on echocardiographic determination of LVH or CT scanning for calcification precludes routine implementation for the purposes of risk stratification in kidney failure [11]. Preliminary data suggest that measures obtained from a routine posterior-anterior (PA) chest X-ray may provide reasonable estimates of vascular calcification and left ventricular size, and could enhance risk prediction without the cost of a CT or echocardiogram [12,13].

Data in the general population has shown aortic arch calcification (AAC) [14,15] and cardiothoracic ratio (CTR) [16-18], both obtained from a routine chest X-ray, to be predictors of CV outcome and mortality, respectively. The prognostic value of these simple measurements has not been studied in kidney failure, but if validated, chest X-ray based measurements could be easily and cheaply implemented with minimal inconvenience to patients and improve risk stratification in the dialysis unit as part of routine clinical care. In many hemodialysis units, chest X-rays are routine for providing information for central line placements, and surveillance for latent tuberculosis.

The objective of this study was to determine whether chest X-ray derived measurements of cardiac size (CTR) and vascular calcification (AAC score), could accurately predict mortality and improve multivariable prognostic models in patients with kidney failure.

## Methods

### Study population

The study was conducted in Winnipeg, Canada and was approved by the research ethics board at the University of Manitoba. We performed a retrospective cohort study utilizing a comprehensive prospective database of all patients initiating dialysis in Manitoba Canada between January 1, 2000 and August 1, 2010 (n = 2368). This database is maintained by the Manitoba Renal Program (MRP), which provides dialysis and chronic kidney disease services for the entire province of Manitoba and areas of Northwestern Ontario (Catchment area approximately 1.5 million). Details of this database have been described in previous studies [19]. Briefly, the database captures patient demographics, cause of ESRD, comorbid conditions, type of dialysis, initial dialysis access, initial blood work, modality transitions within the first 90 days, small molecule clearance, and outcomes such as death, transplantation, or transfer out of province. All new ESRD patients are reviewed in detail at a weekly multidisciplinary team rounds and comorbid conditions recorded in the database by dedicated MRP personnel. All hospitalizations and deaths in the MRP are reviewed and adjudicated weekly at the same team rounds. A subset of this data is forwarded to the Canadian Organ Replacement Register (CORR) maintained by the Canadian Institute for Health Information. For the purposes of the present analysis, we included only adult (>18 years) chronic dialysis (on dialysis >90 days) patients. We examined all-cause mortality as the primary outcome.

### X-ray measurements

Eligible patients identified in the MRP database were linked by PHIN (Personal Health Information Number) and date of birth to a province-wide registry of radiographic procedures (AGFA IMPAX 6) to identify chest X-rays. Inclusion criteria were: initiation of dialysis in Manitoba, Canada between January 1, 2000- Aug. 1, 2010, and the availability of a technically adequate posterior-anterior chest X-ray between the period of three months prior to the initiation of dialysis until death or study end-date. The earliest available (i.e. closest to date of dialysis initiation), technically adequate chest X-ray was chosen for review. We defined technical adequacy as a posterior-anterior chest X-ray exhibiting defined heart borders and a defined aortic knob. Thus, chest X-rays with severe effusions, infiltrates, or anatomic or technique irregularities that precluded identification of cardiothoracic ratio or aortic arch calcification were excluded. Two adjudicators independently assessed technical adequacy, with disagreements resolved by consensus. Both film and digital X-rays were included.

**Figure 1:**
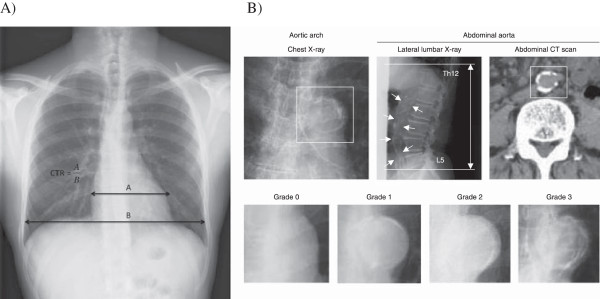
**Clinical assessment of cardiothoracic ratio and aortic arch calcification. A)** Measurement of cardiothoracic ratio (CTR). CTR is equal to the maximal cardiac width divided by thoracic width, as shown. **B)** Assessment of aortic calcification using chest x-ray (lateral lumbar and abdominal CT for comparison with calcification enclosed by white boxes), with examples of grade 0–3 shown. Permission pending for reproduction of this image [12].

The grade of aortic arch calcification was assessed using a previously validated scoring system: grade 0 (no visible calcification), grade 1 (small spots of calcification or single thin calcification of the aortic knob), grade 2 (one or more areas of thick calcification), and grade 3 (circular calcification of the aortic knob) [20]. The cardiothoracic ratio was calculated as the ratio of maximum transverse cardiac diameter in millimeters to maximum thoracic diameter in millimeters. Both AAC grading and CTR measurement are illustrated in Figure 1. All measurements of AAC and CTR were assessed independently by two adjudicators, with disagreements resolved by a consensus measurement.

### Statistical methods

Summary statistics were presented as mean (standard deviation (SD)) or median (25th, 75th centile) as appropriate; categorical values were described as proportions. Univariable comparisons in patient characteristics were performed with analysis of variance (ANOVA) or Chi square test as appropriate.

Univariable Cox proportional hazards regression was used to estimate the unadjusted impact of AAC grade and CTR on all-cause mortality. For these analyses, missing covariate values were imputed using multivariable imputation. In all cases, fewer than 8% of individual covariate values were missing and therefore imputed. Multivariable Cox Proportional Hazards Regression models were constructed to 1) to identify a parsimonious base prediction model (best base model, BBM) using clinical variables alone, 2) to assess whether AAC and CTR were independent of these base model variables in prediction of death, and 3) to calculate the improvement in model discrimination and reclassification after addition of AAC or CTR to the base model. CTR was treated as a continuous variable, and AAC as a categorical variable with 4 levels (0, 1, 2, 3). The base prediction model for death was built from a pool of candidate clinical variables using both statistical and clinical significance; for the purposes of this analysis, variables in the base models were retained either if they were associated with a p < 0.1, or based on known associations with mortality. Two enriched models were created: base plus CTR, and base plus AAC. We assessed model discrimination using Harrell’s concordance statistic (C-statistic) and the integrated discrimination improvement index (IDI). The Harrell’s C statistic corresponds to the area under the receiver-operating curve for the proportional hazards model, and is the standard measure of discrimination. The IDI measures the change in the discrimination slopes between two alternative models, and is considered a more sensitive measure of discrimination than the C-statistic. We also examined model reclassification using the net reclassification index, NRI [21]. NRI measures the ability of a new model to correctly reclassify patients without the outcome of interest (i.e. death) into lower risk categories and patients with the outcome of interest into higher risk categories. For the purpose of the present analysis, we used the following risk classification scheme: high risk, >10% risk of death; moderate risk, 5-10% risk of death; and low risk, <5% risk of death. To be judged clinically useful, the models incorporating AAC and CTR had to exhibit statistically significant improvements in two of the following three measures of predictive model performance: C-statistic, IDI > 10%, and NRI > 10% [22,23]. All statistical calculations were performed using IBM SPSS version 18.

## Results

**Figure 2:**
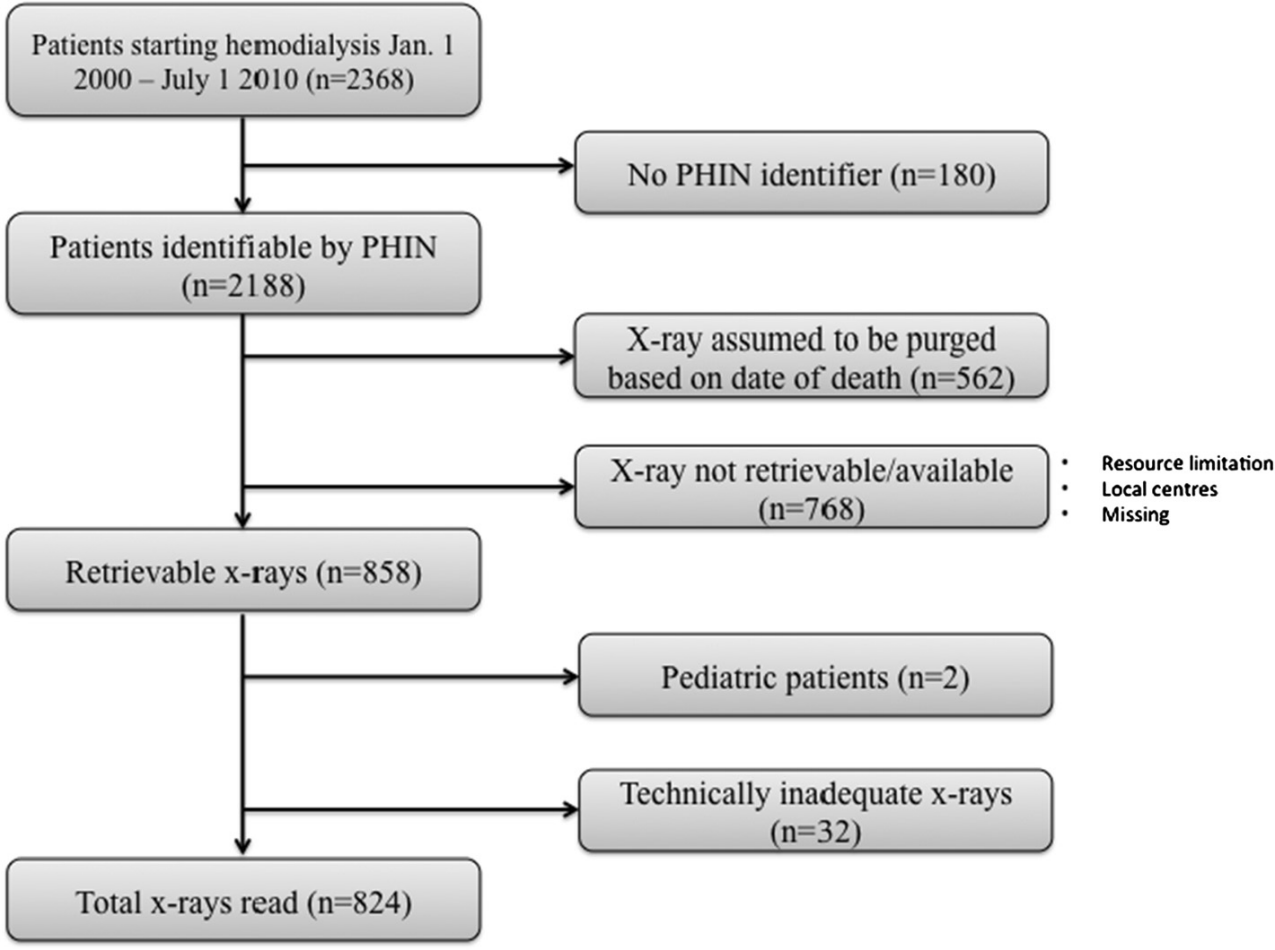
Process of patient exclusion from database.

Of the initial 2368 potentially eligible patients, 824 had technically adequate PA chest X-rays for the study and were included in the analysis. The specific reasons for exclusion are summarized in Figure 2.

### Study population

The baseline characteristics of the study sample are summarized in Table 1. Data on age, sex, diabetic status, race, cause of renal disease, hemoglobin, serum creatinine, serum calcium, serum phosphate, serum albumin, other co-morbidities, and our primary outcome (all-cause mortality) were obtained from the Manitoba Renal Program database. The median age of the cohort was 60 [47, 71] years at the time of X-ray, with a median dialysis vintage of 1.3 years. Fifty-four percent of the cohort was male, and 35% was of aboriginal descent.

**Table 1:** Characteristics of patients with and without an available chest X-Ray

**Characteristics**	**X-ray available**	**X-ray not available**	**p**
Proportion alive at end of follow-up	672/824	444/800	<0.01
Age at dialysis start	57.3 [45.0, 68.1]	63.8 [53.0, 74.8]	<0.01
Age at chest x-ray	59.7 [47.3, 70.7]	-	-
Sex (M)	54%	56%	0.64
Race			
Caucasian	47%	59%	<0.01
Aboriginal	35%	28%	<0.01
Other	18%	13%	0.01
BMI	32.5 [28.5, 38.8]	33.0 [28.2, 38.3]	0.99
Diabetes	25%	20%	0.01
CHF	15%	18%	0.14
Hypertension	67%	70%	0.17
IHD	15%	20%	<0.01
Hemoglobin (g/L)	93 [81, 106]	97 [84, 110]	<0.01
Creatinine (mcm/L)	678 [529, 876]	632 [495, 815]	<0.01
Urea (mmol/L)	31 [25, 40]	32 [24, 39]	0.93
CO_2_ (mmol/L)	20 [16, 23]	20 [17, 24]	0.03
Calcium (mmol/L)	2.1[1.8, 2.3]	2.1 [1.8, 2.3]	0.97
PO_4_ (mmol/L)	2.1[1.7, 2.6]	2.0 [1.6, 2.5]	0.01
Albumin (g/L)	29 [24, 34]	30 [25, 35]	0.01
HD days at chest x-ray	463 [9, 1520]	N/A	-
Follow-up days	1173 [585, 2266]	1112 [453, 1926]	<0.01
Older Vintage (start year < 2005)	29%	30%	0.91
Urban dialysis	83%	68%	<0.01

Of the 824 patients, 152 patients died at a median dialysis time of 2.5 years from initiation. Compared with survivors, patients who died were significantly older (68 years vs. 58 years, p < 0.01) at the chest X-ray date, had a higher prevalence of CHF (22% vs. 14%, p = 0.01), and had been on dialysis longer at the time of X-ray assessment. Median serum creatinine at the start of dialysis (566 vs. 709, p < 0.01) was lower in patients who died (Table 2).

**Table 2:** Characteristics of X-rayed patients, alive and dead

**Characteristics**	**Alive**	**Dead**	**p**
n	672/824	152/824	-
Age at chest x-ray	58.0 [46.1, 68.7]	67.5 [58.7, 78.4]	<0.01
Sex (M)	55%	49%	0.05
Race			
Caucasian	46%	51%	0.35
Aboriginal	34%	38%	0.34
Other	20%	11%	0.02
BMI	32.6 [28.5, 38.8]	31.4 [28.2, 39.4]	0.32
Diabetes	55%	56%	0.90
CHF	14%	22%	0.01
Hypertension	66%	71%	0.24
IHD	14%	20%	0.05
Hemoglobin (g/L)	93 [80, 106]	95 [85, 105]	0.26
Creatinine (mcm.L)	709 [555, 892]	566 [477, 723]	<0.01
Urea (mmol/L)	31 [25, 39]	31 [23, 41]	0.91
CO_2_ (mmol/L)	20 [16, 23]	20 [16, 23]	0.28
Calcium (mmol/L)	2.1 [1.8, 2.3]	2.1 [1.8, 2.3]	0.32
PO_4_ (mmol/L)	2.1 [1.7, 2.7]	2.1 [1.6, 2.5]	0.06
Albumin (g/L)	29 [24, 34]	29 [24, 34]	0.94
HD days at chest x-ray	401 [4.0, 1589]	608 [76, 1327]	0.61
Follow-up days	1257 [626, 2411]	912 [402, 1664]	<0.01
Older Vintage (start year < 2005)	30%	28%	0.77

Median CTR for the cohort was 0.53 [0.48, 0.58], and 67% had a CTR >0.5 (Table 3). The median CTR in patients who died was higher than among survivors (0.55 vs. 0.52, p < 0.01) and 79% had a CTR > 0.5. Overall, 46% had AAC > 0, and among patients who died that proportion rose to 64% vs. 41% in patients who lived.

**Table 3:** Distribution of cardiothoracic ratio and aortic arch calcification grade across study population

**Variable**	**Overall (n = 824)**	**Patients alive at end of follow-up (n = 672)**	**Patients dead at end of follow-up (n = 152)**	**p-value**
**Cardiothoracic ratio (CTR)**	0.53 [0.48, 0.58]	0.52 [0.48, 0.57]	0.55 [0.51,0.59]	<0.001
**CTR > 0.5**	67% (552/824)	64% (527/824)	79% (651/824)	<0.001
**Aortic arch calcification grade (%)**				<0.001
0	54% (445/824)	59% (394/672)	36% (54/152)	
1	24% (198/824)	22% (146/672)	34% (52/152)	
2	17% (140/824)	16% (107/672)	20% (30/152)	
3	5% (41/824)	4% (25/672)	11% (16/152)	

CTR = median [Q1, Q3].

### Comparison with missing data

To assess the possibility of a selection bias, we performed a sensitivity analysis comparing the characteristics of patients with and without an available X-ray (Table 1). On average, patients without X-rays were older, were more likely to be dialyzing outside of our centre, and to be of Caucasian descent. The study population had a higher rate of diabetes than patients without X-rays, but a lower rate of ischemic heart disease. Patients with X-rays also had, on average, higher starting levels of creatinine and phosphate, and lower levels of hemoglobin and serum albumin.

### Risk prediction for all cause mortality

Cardiothoracic ratio (per 0.1 unit change) and aortic arch calcification were both significantly associated with death on univariable proportional hazards analysis (Table 4). Both variables remained statistically significant after multivariable adjustment (Table 5). However, the association between AAC and mortality was significantly attenuated after multivariable adjustment. This attenuation was largely accounted for by confounding with age, as shown in Table 6.

**Table 4:** Relationship between x-ray variables and death (n = 824) – Univariate analyses

**Variables**	**Hazard ratio [95% CI]**	**Model p**	**Model AUROC [95% CI]**
***Model 1***		<0.001	0.60 [0.55, 0.65]
CTR	1.78 [1.40, 2.27]		
***Model 2***		<0.001	0.63 [0.58, 0.68]
AAC	-		
0	referent		
1	2.43 [1.64, 3.61]		
2	2.22 [1.41, 3.49]		
3	4.35 [2.48, 7.66]		
***Model 3***		<0.001	0.65 [0.60, 0.70]
CTR	1.52 [1.17, 1.97]	0.002	
AAC		<0.001	
0	referent		
1	2.16 [1.45, 3.24]		
2	1.81 [1.13, 2.89]		
3	3.49 [1.17, 1.97]		

CTR = Cardiothoracic ratio; AAC = Aortic arch calcification score; AUROC = area under the receiving operator curve.

**Table 5:** Multivariable analysis (n = 824)

**Model and variables**	**Hazard ratio [95% CI]**	**P value**	**Model AUROC [95% CI]**
***Base model (BBM)***		Model P < 0.01	0.71 [0.66, 0.76]
Age at chest x-ray (per year)	1.05 [1.03, 1.06]	<0.01	
Sex	0.91 [0.62, 1.30]	0.62	
Duration of hemodialysis (per year)	0.98 [0.91, 1.01]	0.53	
Race		<0.01	
Caucasian	1.63 [0.90, 2.90]	0.11	
Aboriginal	2.9 [1.5, 5.4]	<0.01	
Other	**referent**	-	
Diabetes	0.71 [0.47, 1.07]	0.10	
Heart failure	1.48 [0.92, 2.39]	0.10	
Ischemic heart disease	1 [0.61, 1.64]	1.00	
Creatinine (per 100 mcmol/L)	1.00 [1.00, 1.00]	0.15	
Phosphate (per mcm/L)	1.0 [0.99, 1.00]	0.38	
***Base model + CTR***		Model P < 0.01	0.71 [0.66, 0.76]
Age at chest x-ray	1.04 [1.03, 1.05]	<0.01	
Sex	1.04 [0.73, 1.45]	0.82	
Duration of hemodialysis	0.97 [0.91, 1.03]	0.33	
Race		0.04	
Caucasian	1.53 [0.89, 2.62]	0.13	
Aboriginal	2.03 [1.16, 3.56]	0.01	
Other	**referent**	-	
Diabetes	0.83 [0.57, 1.20]	0.31	
Heart failure	1.36 [0.91, 2.05]	0.14	
Ischemic heart disease	0.94 [0.60, 1.46]	0.77	
Creatinine	1.00 [1.00, 1.00]	0.14	
Phosphate	1.00 [0.99, 1.00]	0.35	
**CTR**	**1.46 [1.11, 1.92]**	**<0.01**	
**Best base model + AAC**		Model P < 0.01	0.72 [0.67, 0.76]
Age at chest x-ray	1.04 [1.02, 1.05]	<0.01	
Sex	0.97 [0.69, 1.36]	0.86	
Duration of hemodialysis	0.95 [0.89,1.02]	0.14	
Race		0.02	
Caucasian	1.44 [0.84, 2.46]	0.19	
Aboriginal	2.11 [1.20, 3.73]	0.01	
Other	**referent**	**-**	
Diabetes	0.82 [0.57, 1.19]	0.29	
Heart failure	1.47 [0.97, 2.21]	0.07	
Ischemic heart disease	0.85 [0.54, 1.34]	0.48	
Creatinine	1.00 [1.00, 1.00]	0.14	
Phosphate	1.00 [0.99, 1.00]	0.33	
**AAC**		**0.03**	
**0**	**referent**		
**1**	**1.52 [0.99, 2.34]**	**0.06**	
**2**	**1.22 [0.72, 2.05]**	**0.47**	
**3**	**2.49 [1.28, 4.82]**	**0.01**	

CTR = Cardiothoracic ratio; AAC = Aortic arch calcification score; AUROC = area under the receiving operator curve.

**Table 6:** Analysis of confounding: the impact of AAC is dramatically reduced by adjustment for age and other variables

**Variable**	**Unadjusted hazard ratio**	**P value**	**Age-adjusted hazard ratio**	**P value**	Fully adjusted hazard ratio*	**P value**
AAC		<0.001		0.06		0.03
0	referent		referent		referent	
1	2.43 [1.64, 3.61]		1.52 [0.99, 2.33]		1.52 [0.99, 2.34]	
2	2.22 [1.41, 3.49]		1.15 [0.69, 1.92]		1.22 [0.72, 2.05]	
3	4.35 [2.48, 7.66]		2.00 [1.07, 3.75]		2.49 [1.28, 4.82]	

*Adjusted for all variables in the best base model: age at x-ray, race, sex, duration of dialysis, diabetic status, history of heart failure, ischemic heart disease, serum phosphate, and serum creatinine at initiation of dialysis.

The predictive ability of CTR and AAC in addition to our base predictive model for mortality is presented in Table 5. As above, CTR was independently associated with mortality when added to a best base model comprised of the variables age at chest X-ray, sex, duration of hemodialysis, race, diabetes, heart failure, ischemic heart disease, baseline serum creatinine and serum phosphate (predictors of survival in a base Cox model). However, CTR did not significantly increase the c-statistic (i.e. area under the curve of the base model) (0.71 [0.66, 0.76] vs. 0.71 [0.66, 0.76]). Furthermore, it did not improve the IDI (IDI =0) or the net reclassification performance (NRI = 0). Similarly, the addition of AAC to the base model did not improve the IDI or NRI values (see Table 5).

## Discussion

In our study of 824 prevalent patients on hemodialysis, simple measures of cardiovascular risk on plain film chest x-ray (CTR and AAC) did not improve prediction of mortality. While CTR was independently associated with mortality in multivariable survival analysis, it did not consistently improve prediction of mortality risk. Similarly, AAC was associated with mortality after adjustment for potential confounders, but the association was significantly reduced after adjusting for age, and prediction of mortality was not improved. Thus our results do not support the utility of x-ray measures of CTR and AAC for the purpose of predicting mortality in a prevalent hemodialysis cohort.

Previous studies in non-ESRD patients have shown that extent of vascular calcification reported from a chest X-ray is strongly associated with mortality, cardiac events, as well as coronary, abdominal aortic, and other vascular calcification [12,15]. Furthermore, vascular calcification beyond the thoracic aorta has been shown to be associated with mortality in ESRD patients [12,24]*.* Using the grading system described previously, AAC detectable on chest X-ray has been shown to be a strong independent predictor of new CV events beyond traditional risk factors [14]. In a study of 401 incident patients on dialysis in Japan, only a borderline significant association between AAC and CV mortality (but not all cause mortality) was identified [25]. While our study found broadly similar results, we examined all cause mortality in a prevalent cohort with a larger sample size. Moreover, our study formally assessed the ability of AAC to improve the accuracy of a predictive model incorporating standard clinical variables. Statistical significance in a multivariable model does not automatically guarantee improvements in discrimination and reclassification compared to standard clinical variables alone, a point well illustrated by our results. Although AAC remained statistically significant after multivariable adjustment, addition of AAC to a base clinical model did not significantly improve prediction. Finally, we observed that AAC was strongly confounded by age. Adjustment for age was responsible for most of the attenuation of the association between AAC and death in the multivariable models (Tables 5 and 6).

Evidence in the general population supports CTR as a predictor of mortality. A high CTR is an indicator of an enlarged heart and is a predictor of poor outcome in heart failure patients [16-18]. In the ESRD population, the evidence is less clear. In a study of 468 hemodialysis patients in Taiwan, CTR predicted both all-cause and CV mortality at 2-years [26]. While our results also showed an independent association between CTR and all cause mortality on multivariable analysis, we additionally examined whether CTR could improve the discrimination and reclassification of a base clinical prediction model. As with our analysis of AAC, we were unable to show improved prediction for all-cause mortality (IDI and NRI = 0).

Strengths of the study include it’s cohort design, large sample size, and analytic strategy. The prospect of a simple, inexpensive, routine imaging modality held promise as a cheap method of risk assessment. Our study population was unique in its size, its large aboriginal representation, and that all patients received dialysis through a unified program (Manitoba Renal Program). Another strength was the independent review process for CTR and AAC. Taking the mean of two independent CTR measurements per X-ray ensured precision, while settling all AAC discrepancies by consensus achieved the same result.

Our study also has several limitations that must be kept in mind when interpreting the results. First, our study had high rate of exclusions due to absent x-rays. These exclusions are partly explained by the fact that patients without a chest X-ray were more likely to receive dialysis in a rural, satellite setting, where acquisition of a chest X-ray is improbable in the context of this study. Nevertheless, by analyzing a non-random subset (i.e. patients with available x-rays) of younger patients with a lower rate of IHD, we may have underestimated the true association between CTR/AAC and mortality.

Second, the historical nature of our cohort imposes additional limitations. Our database did not include information on cause specific mortality. As CTR and AAC are causally associated with CV causes of death, using all cause mortality rather than CV mortality as the outcome could have weakened the observed association between these variables and outcome. On the other hand, from a clinical perspective, all cause mortality is the outcome of most relevance to clinicians and patients. Even had we been able to demonstrate a predictive value of AAC or CTR for CV mortality, the impact of these findings would have been attenuated in the absence of a demonstrated predictive value for all cause death. Third, we were unable to ascertain the timing of x-rays in relation to a dialysis run or to dry weight. The CTRs measured therefore represent a combination of volume expansion and LV mass. However, as both volume overload and LV mass are associated with death, it is unlikely that this confounding attenuated the observed association between CTR and death. Finally, since we analyzed a prevalent cohort, a survivor bias may be present which may additionally have attenuated the risk factor-outcome associations.

## Conclusions

In summary, our data do not support the clinical utility of simple plain X-ray measures of cardiac size and vascular calcification for the purpose of mortality prediction in maintenance hemodialysis patients. More advanced imaging techniques such as cardiac MRI and coronary CT may be needed to improve mortality prediction in this population.

### Consent

Given the retrospective nature and low privacy risk of the protocol, the need for patient consent was waived by the University of Manitoba health research ethics board.

## Competing interests

We have no conflicts of interest to report. The results presented in this paper have not been published previously in whole or part, except in abstract form.

## Authors’ contributions

EB was involved in study conception, data collection, analysis of data, and drafting/revision of article. NT participated in drafting/revision of article. BG was involved in data collection, and data analysis. BH was involved in study conception, and revision of the manuscript. MMS was involved in revision of the manuscript. PK was involved in study conception, and the revision of the manuscript. CR was involved in study conception, analysis of data, and drafting/revision of the manuscript. All authors read and approved the final manuscript.

## Pre-publication history

The pre-publication history for this paper can be accessed here:

http://www.biomedcentral.com/1471-2369/14/263/prepub
